# SNPraefentia: a toolkit to prioritize microbial genome variants linked to health and disease

**DOI:** 10.1093/bioadv/vbaf297

**Published:** 2025-11-22

**Authors:** Nadeem Khan, Muhammad Muneeb Nasir, Ammar Mushtaq, Masood Ur Rehman Kayani

**Affiliations:** Metagenomics Discovery Lab, School of Interdisciplinary Engineering & Sciences (SINES), National University of Sciences & Technology (NUST), Islamabad 44000, Pakistan; Metagenomics Discovery Lab, School of Interdisciplinary Engineering & Sciences (SINES), National University of Sciences & Technology (NUST), Islamabad 44000, Pakistan; Computational Aeronautics Lab, School of Interdisciplinary Engineering & Sciences (SINES), National University of Sciences & Technology (NUST), Islamabad 44000, Pakistan; Metagenomics Discovery Lab, School of Interdisciplinary Engineering & Sciences (SINES), National University of Sciences & Technology (NUST), Islamabad 44000, Pakistan

## Abstract

**Motivation:**

Analysis of genomic variation in microbial genomes is crucial for understanding how microbes adapt, interact with their hosts, and influence health and disease. In metagenomic studies, where genetic material from entire microbial communities is sequenced, thousands of single-nucleotide polymorphisms can be detected across species and samples. However, identifying which of these variations has biologically or functionally relevant impacts remains a significant challenge.

**Results:**

To address this, we present SNPraefentia, a Python-based toolkit for prioritizing microbial SNPs based on their predicted functional relevance. The tool integrates multiple biologically meaningful parameters, including sequencing depth, physicochemical impact of amino acid substitutions, and the structural and functional context of mutations within annotated protein domains. SNPraefentia extracts variation depth and amino acid changes, annotates protein domains using UniProt, and computes individual impact scores. These are then integrated into a composite prioritization score that reflects the potential biological importance of each variant. Overall, SNPraefentia provides researchers with a systematic and reproducible approach to filter and rank microbial variants for downstream functional analysis or experimental validation.

**Availability and implementation:**

The toolkit and test data are freely available at https://github.com/muneebdev7/SNPraefentia.

## 1 Introduction

Single-nucleotide polymorphisms (SNPs) in microbial genomes are key drivers of evolutionary change, phenotypic diversity, and functional adaptation. In clinical and ecological contexts, specific SNPs can influence antimicrobial resistance, virulence, host interaction, and metabolic capabilities. Accurately identifying and prioritizing these SNPs (further referred to as variants) is essential for understanding microbial behavior and guiding downstream experimental analyses (Riccardi and Resta 2020, Li *et al.* 2021, Miller and Chiu 2021, Zhang and Knight 2023, Bhunia *et al.* 2024, Linz *et al.* 2024). Several tools, such as SnpEff and Snippy, are currently deployed for detecting and annotating variants in microbial genomes (Seemann 2015). However, with the increasing complexity of microbial genomic datasets, owing to the advances in genome-resolved metagenomics and strain-resolved analyses, there is an urgent need for more advanced methods that go beyond identification and annotation and enable prioritization of variants relevant to health and disease.

To address this gap, we present SNPraefentia, a tool developed for prioritizing variants in microbial genomes. Unlike currently available variant prioritization tools, designed for human datasets, SNPraefentia is tailored for microbial systems (Gong *et al.* 2015, Yauy *et al.* 2018). It incorporates multiple biologically significant features, including variant read depth, changes in amino acid physicochemical properties (e.g. size, charge, hydrophobicity, polarity), and the presence of mutations in known functional protein domains. The integration of these features yields a composite prioritization score that reflects the cumulative biological importance of each variant. This framework enables users to rank SNPs based on their potential to influence microbial function and clinical relevance. By automating this process, SNPraefentia empowers researchers to focus on high-impact variants, facilitating more targeted studies across diverse microbial genomes, including those identified or reconstructed from metagenomic samples.

## 2 Tool description

**Figure 1.**
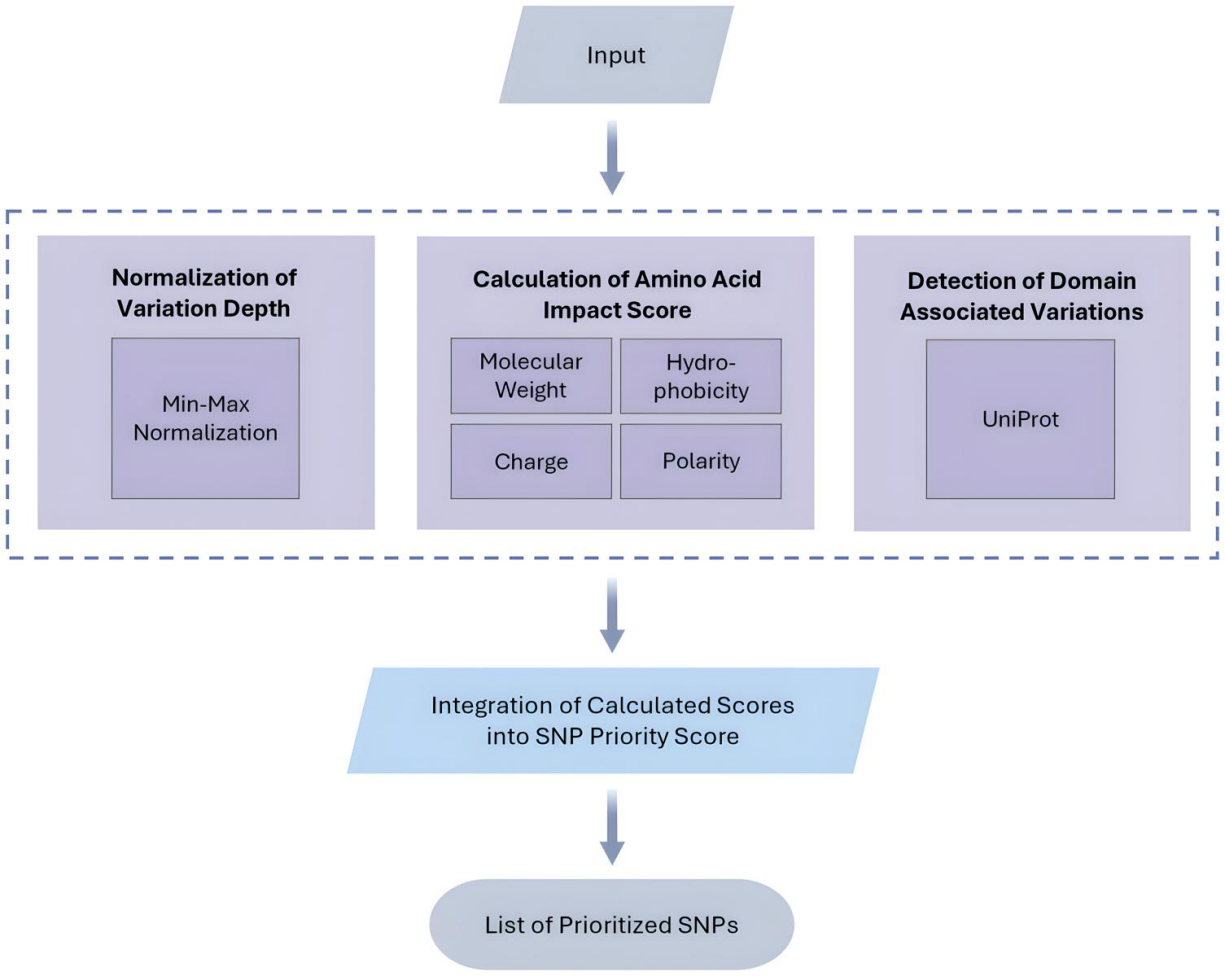
The workflow of SNPraefentia. The tool processes an input file of annotated variants. It separately calculates scores considering three key modules: variation depth, amino acid impact score, and presence of variation in the functional domain of the protein. These scores are consolidated to generate a final priority score for each variant.

SNPraefentia is implemented in Python and developed as a command-line tool for transparency, modularity, and ease of use. The tool is packaged and publicly available through the Python Package Index (PyPI) and can be installed using the following command: *pip install snpraefentia*. The source code is publicly available at https://github.com/muneebdev7/SNPraefentia. The overall architecture of SNPraefentia is shown in Fig. 1, outlining the sequential flow from parsing the input to the generation of a prioritized variant list.

### 2.1 Input file

SNPraefentia accepts variant annotations in tab-separated (tsv) or comma-separated (csv) formats (Supplementary Table 1, available as supplementary data at *Bioinformatics Advances* online) as input, typically generated from a variant calling pipeline (e.g. Snippy). The input file must contain the following four columns: (i) Gene, that indicates the microbial gene in which the variation has been identified; (ii) Amino_Acid_Position, representing the genomic variant position in the format x/y where x is the variation site and y is the total number of amino acids in the protein; (iii) Effect, describing the substitution at amino acid level (e.g. Val170Leu); and (iv) Evidence, indicating the variant depth.

### 2.2 Normalization of variant read depth

Variant read depth is one of the key metrics in prioritizing variants, since high-depth variants are more likely to represent true biological signals rather than sequencing noise (Jee *et al.* 2016, Farheen *et al.* 2017). To make depth values comparable across samples or genes with varying coverage, SNPraefentia applies min–max normalization (also referred to as min–max scaling) to transform variant read depth values into a standard range of [0,1]. This approach was selected purely for scaling purposes, as all input variants had already met the quality and depth thresholds defined by Snippy, ensuring the absence of false variant calls. The resulting scaled values enable consistent integration into the final prioritization score without distorting their intrinsic relationships. The normalized depth (*D*_normalized_) is calculated as shown in (1).


(1) $$D_{\mathrm{normalized}}=\frac{D_{i}-D_{\min}}{D_{\max}-D_{\min}}$$

In this equation, *D_i_* is the observed variant read depth for the *i*th SNP; *D*_min_ is the minimum depth across all SNPs in the dataset; *D*_max_ is the maximum depth across all SNPs in the dataset; and *D*_normalized_ is the scaled depth value between 0 and 1.

This normalization ensures that all depth values contribute proportionally when integrated into the final prioritization score.

### 2.3 Calculation of amino acid impact score

Amino acid impact score is calculated by integrating four biologically meaningful properties of amino acids: (i) molecular weight, (ii) hydrophobicity, (iii) polarity, and (iv) charge. The differences in molecular weight (*W*_diff_) and hydrophobicity (*H*_diff_) are normalized, according to (2) and (3), using the maximum observed ranges of these properties across natural amino acids: 204 Da (tryptophan) to 75 Da (glycine) for weight (range ≈ 130), and −4.5 to +4.5 for hydrophobicity (range = 9). Polarity and charge differences are treated as binary indicators (0, 1). These four features are integrated (4) into a unified value (amino acid impact score), reflecting the degree of physicochemical disruption introduced by the variation.


(2) $$W_{\mathrm{diff}}=\frac{\left|W_{\mathrm{reference}}-W_{\mathrm{mutated}}\right|}{130}$$

(3) $$H_{\mathrm{diff}}=\frac{\left|H_{\mathrm{reference}}-H_{\mathrm{mutated}}\right|}{9}$$

(4) $$\mathrm{Amino}\;\mathrm{Acid}\;\mathrm{Impact}\;\mathrm{Score}=W_{\mathrm{diff}}+H_{\mathrm{diff}}+{P_{\mathrm{change}}+C}_{\mathrm{change}}$$

Here, *W*_reference_ and *W*_mutant_ denote the molecular weights of the original and substituted amino acids. *H*_reference_ and *H*_mutant_ represent respective hydrophobicity values. *P*_change_ is 1 if the polarity changes between the two residues, otherwise 0. *C*_change_ is 1 if the charge differs, otherwise 0.

### 2.4 Detection of domain-associated mutations

SNPraefentia determines whether a mutated amino acid resides within a known functional domain by querying domain annotations from the UniProt database. Gene, Taxonomic_ID, and Total_Protein_Length are used to identify the closest matching UniProt entry (UniProt_ID) via the UniProt REST API. If the variant residue falls within the functional domain region of the protein, the variant is assigned a score of 1; otherwise, 0. This binary feature, termed Domain_Position_Match, prioritizes variants that are likely to affect functionally relevant regions of microbial proteins.

To minimize potential biases from assembly errors or annotation inconsistencies, SNPraefentia, as mentioned, applies a careful matching strategy that cross-references the bacterial species, gene identity, and total protein length to ensure accurate UniProt mapping. In cases where reliable domain information is unavailable, a neutral score of 0 is assigned instead of an inferred value, preventing artificial inflation of the prioritization score. Moreover, although domain mapping depends on UniProt annotations, the tool remains applicable to novel or less-characterized microbial species, like when domain data are missing, SNPraefentia integrates five additional biological features, each contributing to the final prioritization score. This design ensures stable and generalizable variant prioritization across both well-annotated and newly reconstructed microbial genomes.

### 2.5 Calculation of final priority score

The final priority score is a composite metric derived by integrating all relevant features into a single value (5). The scoring formula assigns double weight to the normalized depth, reflecting the increased confidence in high-frequency mutations, while the amino acid impact score and Domain Position Match are weighted equally. The total score is scaled by dividing by 7, the maximum possible sum of all weighted components, and multiplying it by 100, ensuring the output remains in the range of 0–100.


(5) $$\mathrm{Final}\;\mathrm{Priority}\;\mathrm{Score}\;(\%)=\frac{2*D_{\mathrm{normalized}}+\mathrm{Amino}\;\mathrm{Acid}\;\mathrm{Impact}\;\mathrm{Score}+\mathrm{Domain}\;\mathrm{Position}\;\mathrm{Match}}{7}*100$$

Variants with higher scores are ranked as higher priority candidates for downstream characterization and experimental validation.

## 3 Working principle of the tool

For the demonstration, we processed an input dataset of 844 variants (Supplementary Table 1, available as supplementary data at *Bioinformatics Advances* online) using SNPraefentia. For successful execution of the analysis, users must provide an input file, the name of the target bacterial species (e.g. *Bacteroides uniformis*), and the output file. The—*input* or *-i* argument specifies the SNP file, while the—*specie* or *-s* argument is used for specifying the corresponding bacterial species. Additionally, the—*output* or *-o* argument defines the output file (Supplementary Table 2, available as supplementary data at *Bioinformatics Advances* online), which provides a comprehensive prioritization of input variants based on the mentioned parameters. The complete execution and progress of SNPraefentia during analysis is shown in Supplementary Fig. 1, available as supplementary data at *Bioinformatics Advances* online.

The primary output of SNPraefentia is the Final_Priority_Score, which serves as the core outcome of the tool, allowing users to quickly identify high-priority variants for downstream analysis. The score is derived from three supporting columns: Normalized_Depth, Amino_Acid_Impact_Score, and Domain_Position_Match, all of which are computed based on the input data and external annotations as described previously.

**Figure 2.**
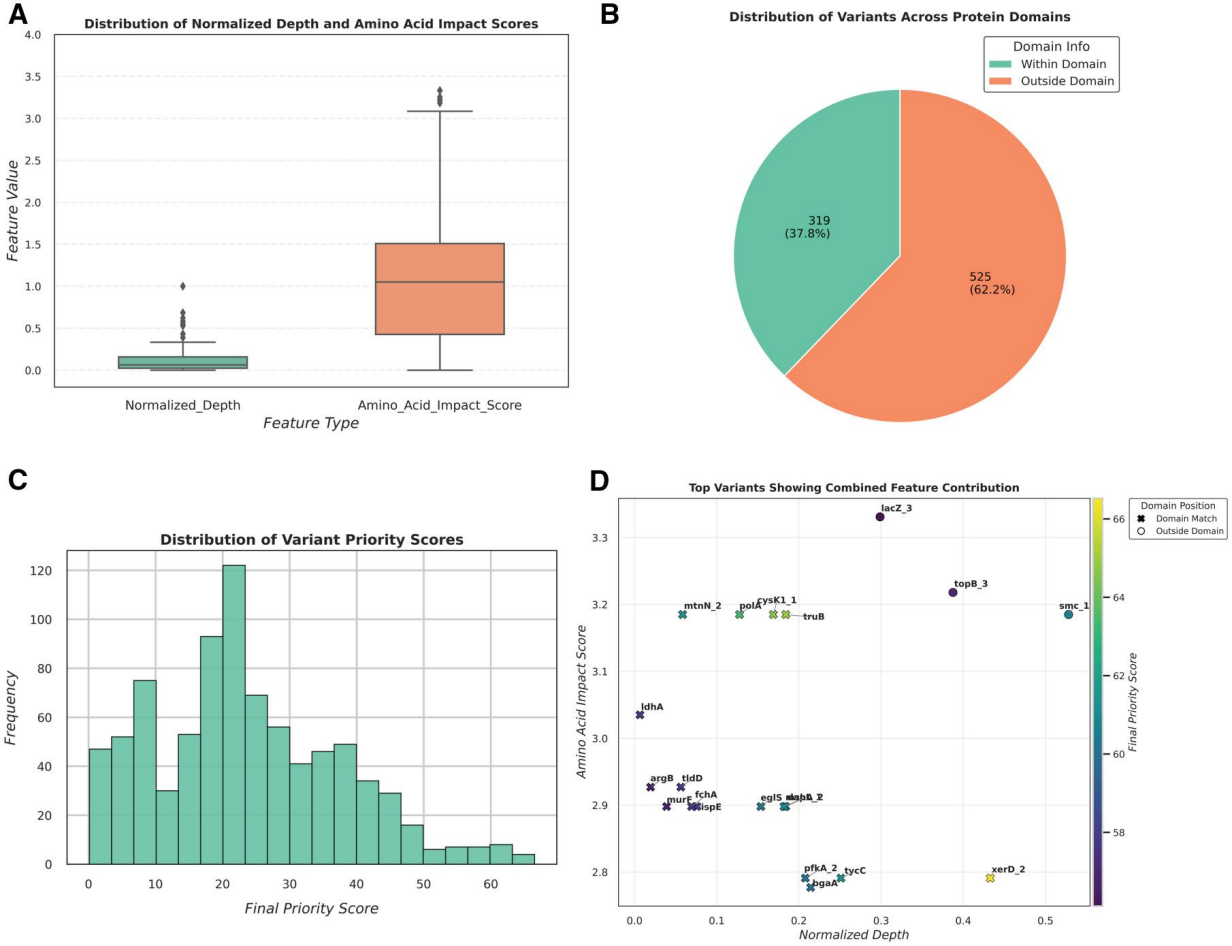
Graphical output generated by SNPraefentia. (A) Box plots showing the distribution of “Normalized_Depth” and “Amino_Acid_Impact_Score” across all variants. (B) Pie-chart illustrating the proportion of variants located within and outside conserved domains. (C) Histogram representing the distribution of “Final_Priority_Score” across the dataset. (D) Scatter plot integrating all feature contributions, displaying the relationship between “Normalized_Depth” (*x*-axis), “Amino_Acid_Impact_Score” (*y*-axis), “Domain_Position_Match” (point shape), and “Final_Priority_Score” (color intensity). Gene names are labeled for the top variants with the highest scores.

To provide a visual overview of these prioritization parameters, a comprehensive set of graphical summaries is generated by SNPraefentia (Fig. 2). The box plots display the overall distribution of Normalized_Depth and Amino_Acid_Impact_Score, enabling users to assess the variability and range of these continuous features. The pie chart depicts the proportion of variants occurring within and outside conserved domains, highlighting the structural relevance of prioritized mutations. The histogram illustrates the frequency distribution of variants across different Final_Priority_Score ranges, giving a clear view of the overall scoring landscape. Finally, the scatter plot integrates all feature contributions, visualizing how depth, impact score, and domain alignment collectively influence the final prioritization, with the top 20 variants and their corresponding genes annotated for user interpretation.

## Supplementary Material

vbaf297_Supplementary_Data

## Data Availability

The toolkit and test data are freely available at https://github.com/muneebdev7/SNPraefentia.
