# Supplementary material for: SNPraefentia: a toolkit to prioritize microbial genome variants linked to health and disease
Source: Bioinform Adv. 2025 Nov 22;5(1):vbaf297. doi: 10.1093/bioadv/vbaf297 (PMC12671963; doi:10.1093/bioadv/vbaf297)
Supplement: vbaf297_Supplementary_Data [file vbaf297_supplementary_data.zip › Supplementary Figures.docx]

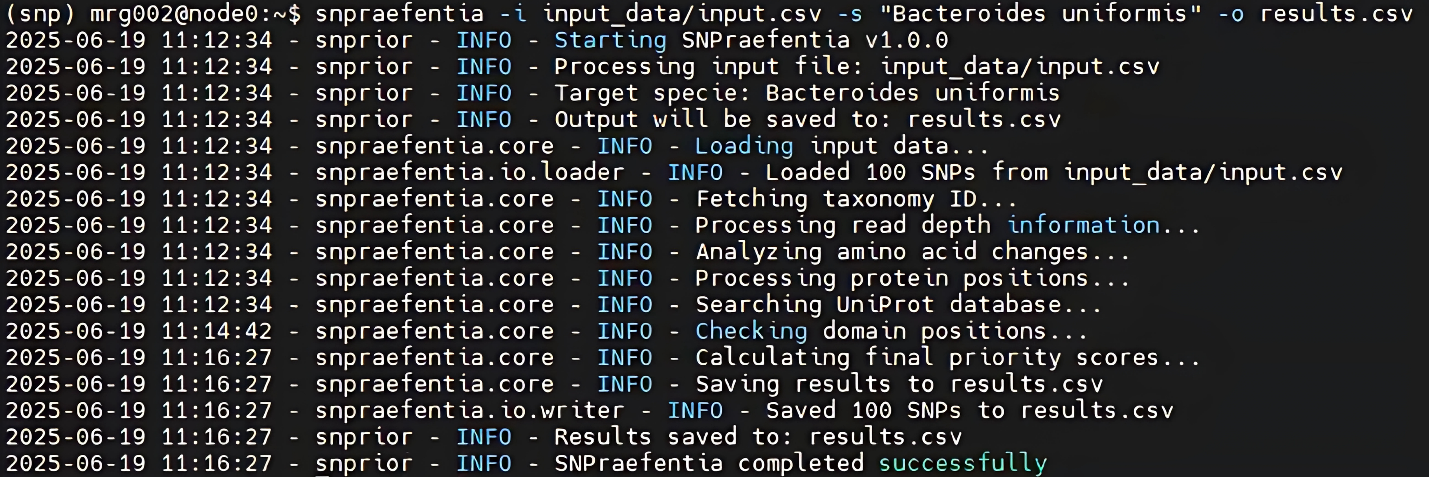


**Supplementary Figure 1:** Command-line execution of SNPraefentia. The user provides the input file using *-i*, specifies the target taxon with *-s*, and defines the output name with *-o*. During execution, SNPraefentia displays status messages to inform the user of its progress. Upon successful completion, a confirmation message is shown.
